# Sharing clinical trial results with participants: important, expected and possible

**DOI:** 10.1186/s13063-025-08802-0

**Published:** 2025-03-27

**Authors:** Annabelle South, Claire Bale, Enhad Chowdhury, Alexander Churchill, Kim Donoghue, Simon Grieveson, Charlotte Hartley, Catriona Manville, Angela Polanco, Kieran Prior, Sheona Scales, Kirstie Shearman, Mahesh K. B. Parmar

**Affiliations:** 1 MRC CTU at UCL, Institute of Clinical Trials and Methodology, UCL, London, UK; 2 Parkinson’s UK, London, UK; 3 Versus Arthritis, London, UK; 4 Department of Health and Social Care, London, UK; 5 Wellcome Trust, London, UK; 6 Prostate Cancer UK, London, UK; 7 Association of Medical Research Charities, London, UK; 8 National Institute for Health and Care Research, London, UK; 9 Cancer Research UK, London, UK; 10 Alzheimer’s Research UK, Cambridge, UK; 11 Health Research Authority, London, UK

**Keywords:** Research ethics, Transparency, Feedback of results, Communicating results, Researcher-participant relations, Trial conduct, Trial ethics

## Abstract

Participants in clinical trials should be pro-actively offered the results of trials in which they have participated. This should be done in a way that is accessible and understandable for all participants. People who have taken part in research have a right to know the results of the studies in which they have taken part and should be given the option of receiving these results. Most trial participants want to receive the overall results. Key reasons for sharing results include respecting participants’ contributions, enhancing their understanding of research benefits, increasing transparency, and potentially improving recruitment and retention. We propose 8 principles to guide sharing of results with trial participants:

1. Trial teams should pro-actively offer overall study results to all clinical trial participants, irrespective of what the results show.

2. Participants should be given the choice of whether to receive research results.

3. Results should be offered to participants in a timely manner.

4. Trial teams should manage participants’ expectations around when the results will be available.

5. Results should be offered in a way that is accessible to participants, both in terms of the communication mechanism and the content.

6. Patient and public involvement is essential in sharing results with participants.

7. Sharing results with participants requires resources.

8. Consideration needs to be given to potential barriers/challenges to sharing results with participants from the planning stage of the study.

Participants in clinical trials should be pro-actively offered the results of trials in which they have participated. This should be done in a way that is accessible and understandable for all participants. Most trial participants never get to find out the results they have contributed to [1]. We believe this is unacceptable. This topic is timely given the forthcoming clinical trials legislation in the UK, which will introduce a legal requirement to offer to share trial findings with participants in a timely way, and in language which they can understand [2, 3]. It is also a priority area for the UK Health Research Authority as part of their research transparency strategy [4].

This Letter is the output from a workshop on sharing clinical trial results with participants, held in June 2024, attended by the authors of this article. Together, our organisations fund, support and regulate health and social care research. The views expressed in this article are not government policy. This Letter focuses on communicating the results of clinical trials to participants. However, the principles it contains may be of broader relevance to other types of clinical research studies.

## Why trial participants should be offered the results

People who have taken part in trials have a right to know the results of the trials in which they have taken part [5]. They should be given the option of receiving these results [6]. Scientific progress is dependent on the contributions of research participants. Without people agreeing to take part in trials, we would not be able to find out whether new treatments, diagnostics and prevention approaches work. Most trial participants want to receive the overall results [7, 8].

Offering results to trial participants:

- Demonstrates respect for their contribution [9].
- Helps participants understand the benefits of research [10].
- Increases research transparency and helps tackle mistrust of researchers [10].
- May encourage better recruitment and retention [11, 12]. (For some, knowing they will receive trial results is a motivation for taking part [8]. Receiving results may inspire participants to take part in future trials, and encourage friends and families to as well).
- Helps participants process their trial experiences [9].

## Principles for sharing results with participants

At the workshop we developed 8 principles for sharing results with participants. Where these are based on specific pieces of research, we have cited the study. Other principles were based on discussion at the workshop. Researchers should apply these principles when sharing results with participants:

1. Trial teams should pro-actively offer overall study results to all clinical trial participants. The research is not finished until participants have had a chance to learn the results.
  Results should be offered irrespective of what they show [8].
  If a trial has been stopped early, results should still be offered to participants. If not available, information on why should be provided; this needs to be done sensitively.
  Where consent is given by a carer/guardian rather than the participant themselves, it may be appropriate to offer results to the person who provided consent in addition to/instead of the participant themselves. Where the participant has capacity, their preferences should be consulted.
  Putting the results on a trial database or webpage and expecting participants to find them based on links given in the patient information sheet is not usually sufficient.
  If the trial has multiple stages or timepoints for results, it may be appropriate to share results as the study progresses to keep participants engaged and informed.
  Careful thought should be given to whether and, if so, how, to offer trial results to loved ones of trial participants who have passed away during or since the trial. This is a sensitive issue, which has potential to cause distress, but may be important for some people [13]. Consideration of ethical and data protection issues, including whether the participant consented to their loved one being informed of trial results, is essential.
2. Participants should be given the choice of whether to receive trial results [8]. While most will want them, not all will and they should be given the opportunity to decide this and change their minds if they want to.
3. Results should be offered to participants in a timely manner [14, 15]. Where feasible, those who have taken part in the trial should be notified as soon as results are publicly available, ideally in parallel with the announcement of results to scientific audiences. The proposed UK clinical trials legislation specifies results should be offered to participants within 12 months of the conclusion of the trial [2].
4. Trial teams should manage participants’ expectations around when the results will be available [15]. This includes giving an approximate timescale in the patient information sheet and providing updates if this timeline changes significantly.
5. Results should be offered in a way that is accessible to participants, both in terms of the communication mechanism and the content (language, images) [8, 15].
  This means not simply sharing information developed for scientific or clinical audiences (although this should be available too, for those who want it, where possible).
  Consideration should be given to equity issues around how results are shared, including the communication mechanism and language(s) used [16].
  Offering participants a choice in how to receive results may, if feasible, help address the different needs and preferences of different participants [15].
6. Patient and public involvement is essential in thinking about, planning and preparing the content to be shared with participants [15, 17].
7. Sharing results with participants requires resources [9]; these should be accounted for in funding calls and applications [17]. Research funders, institutions and research teams have a duty to make sure the resources needed for sharing results with participants are planned and will be available when needed. This can be challenging, given the competing demands on limited research funding, and the need to adapt plans to respond to unexpected results or changes in circumstances. When trial results are not shared with participants, it reflects badly on both researchers and research funders. Mechanisms should be considered including funding through trial grant applications or a centralised pot to which researchers can apply should results communication need to take place after trial funding has ended. Time required for sharing results with participants should be included in agreements with sites where sites are expected to do this.
8. Consideration needs to be given to potential barriers/challenges to sharing results with participants at the planning stage of the study to allow these barriers/challenges to be identified and addressed [15]. Barriers may include lack of prioritisation, challenges communicating complex science to lay audiences, resources required, challenges communicating sensitive results, misconceptions around the ethics approvals required, other logistical issues and the risk of reinforcing inequalities. We have produced guidance on this topic, based on the workshop discussions [16].

## Data Availability

Not applicable.
